# Washing with alkaline solutions in protein A purification improves physicochemical properties of monoclonal antibodies

**DOI:** 10.1038/s41598-021-81366-6

**Published:** 2021-01-19

**Authors:** Yuichi Imura, Toshiaki Tagawa, Yuya Miyamoto, Satoshi Nonoyama, Hiroshi Sumichika, Yasuhiro Fujino, Masaya Yamanouchi, Hideo Miki

**Affiliations:** 1 Sohyaku. Innovative Research Department, Mitsubishi Tanabe Pharma Corporation, Yokohama, Japan; 2 Sohyaku. Innovative Research Department, Mitsubishi Tanabe Pharma Corporation, Fujisawa, Japan; 3 Present Address: Development Department, Tanabe Research Laboratories U.S.A. Inc., San Diego, USA; 4 Present Address: Research Department, Tanabe Research Laboratories U.S.A. Inc., San Diego, USA

**Keywords:** Proteins, Antibody therapy, Analytical chemistry

## Abstract

Protein A affinity chromatography has been widely used for both laboratory scale purification and commercial manufacturing of monoclonal antibodies and Fc-fusion proteins. Protein A purification is specific and efficient. However, there still remain several issues to be addressed, such as incomplete clearance of impurities including host cell proteins, DNA, aggregates, etc. In addition, the effects of wash buffers in protein A purification on the physicochemical characteristics of antibodies have yet to be fully understood. Here we found a new purification protocol for monoclonal antibodies that can improve physicochemical properties of monoclonal antibodies simply by inserting an additional wash step with a basic buffer after the capture step to the conventional protein A purification. The effects of the alkaline wash on monoclonal antibodies were investigated in terms of physicochemical characteristics, yields, and impurity clearance. The simple insertion of an alkaline wash step resulted in protection of antibodies from irreversible aggregation, reduction in free thiols and impurities, an improvement in colloidal and storage stability, and enhanced yields. This new procedure is widely applicable to protein A affinity chromatography of monoclonal antibodies.

## Introduction

Since the first therapeutic monoclonal antibody OKT-3 was approved by the FDA, approximately 80 monoclonal antibodies have been approved for therapeutic uses so far^1^. Antibody therapeutics are essential in medical care, especially in the field of autoimmune diseases and cancer^1^. In addition to the high target specificity of these molecules, their long in vivo half-life conferred by the Fc region renders them ideal for therapeutic uses^2^.

Besides pharmacokinetics, the Fc region plays an important role in purification of antibodies. Protein A derived from *Staphylococcus aureus* strongly binds to the Fc region^3^. Therefore, affinity columns consisting of immobilized protein A and engineered variants have been widely used for laboratory scale purification and commercial manufacturing of Fc-containing proteins^4–6^. Although these protein A columns can yield highly purified Fc-containing proteins in a single step, there are several problems to overcome, i.e., low yield with conventional acid elution for some antibody clones^7,8^ and incomplete clearance of several impurities such as fragments, aggregates, host-cell-derived proteins and nucleic acids etc^4,9^.

Especially, aggregation is one of the big concerns in the development of protein pharmaceuticals because aggregated proteins are considered a likely cause of immunogenicity which can lead to severe adverse events^10–12^. Therefore, aggregation should be minimized for protein pharmaceuticals. To minimize aggregation of therapeutic antibodies, several attempts have been made at each step of drug development; i.e. antibody engineering^13^, extensive developability assessment^14^, process development^15^, and formulation optimization^16^.

Previous studies reported that the protein A purification step itself could increase aggregation^9,17,18^. Aggregates generated during protein A purification are typically removed by successive orthogonal chromatographies such as hydrophobic interaction chromatography and ion exchange chromatography^6^. Improving the impurity clearance of the protein A step may reduce the required subsequent purification steps of the therapeutic antibody and increase yield.

Studies on optimisations of protein A purification have been carried out by many groups so far. For example, some groups focused on optimization of elution buffers (pH, arginine, buffer components etc.) to minimize aggregation and maximize recovery^7,17^.

Other studies tried to improve the washing step after the capture step. Effects of washing buffers containing urea^19,20^, organic solvents^19,20^, detergents^19,20^, high salt^20–22^, polymers^20^, arginine^21,22^, guanidine^21^, and basic pH^19,22,23^ were explored. However, the main purpose of these studies was limited to reducing the residual host cell proteins as well as improving recoveries. Therefore, a detailed analysis was not carried out and the effects of wash buffers on the physicochemical properties of antibodies were not fully elucidated.

**Figure 1:**
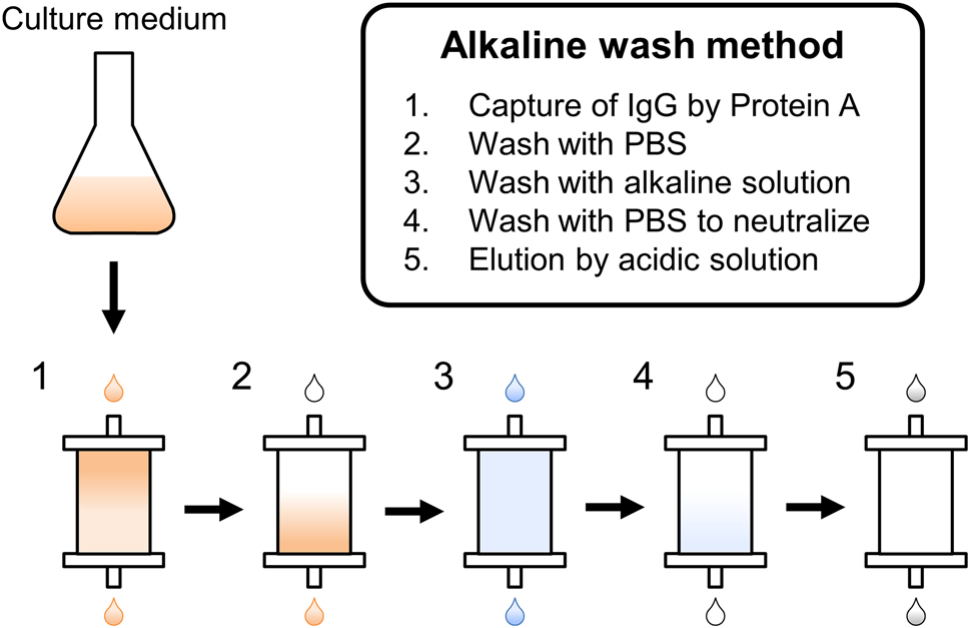
Schematic workflow for purifying antibody on Protein A with alkaline wash. (1) Culture medium was loaded onto the Protein A column equilibrated with PBS. The column was washed with (2) PBS and (3) alkaline solution (pH 11.0, unless otherwise indicated). (4) The column was then washed with PBS to adjust neutral pH. (5) Finally, the antibody was eluted with acidic solution.

Although a yield of an antibody in protein A purification is often relatively high, some antibodies show low yields^7,8^. We encountered some antibodies with low yields when purified by a standard protocol. During our efforts to optimize purification protocols for these antibodies, we serendipitously found that a wash step of antibodies bound to protein A column with alkaline buffers (pH > 10) improved physicochemical characteristics, recoveries and residual host cell proteins (Fig. 1)^24^. In this study, several physicochemical analyses were carried out to elucidate the effects of the alkaline wash procedure during protein A purification. The alkaline wash method protected antibodies from irreversible aggregations possibly by lowering the amount of free thiols, and improved their colloidal and short-term stabilities. Yields of these antibodies were also improved. This new purification method will contribute to protein A purification for both laboratory scale and manufacturing scale by reducing purification steps and time for process development.

## Materials and methods

### Antibody preparation

Seventeen IgG1 λ and 2 IgG1 κ antibodies used in this study were expressed by the commercially available mammalian expression systems; Free Style 293-F (Life Technologies) and CHO K1SV GS system (Lonza) were used for the transient and stable expression, respectively. The cultured medium expressing IgG was prepared based on the standard procedure provided by the manufacturers.

The expressed antibodies were purified by the chromatography system AKTA explorer 100 (GE Healthcare). The protein A column with the column volume (CV) of 5 mL (HiTrap MabSelect SuRe, GE Healthcare) was connected to the purification system. The pH working range of the column is from 3 to 12^25^. After equilibrating the column with PBS pH 7.2 (Life Technologies), the cultured medium was applied to the system. The flow rate was maintained at CV/min. The volume of cultured medium in one purification batch was in the range of 480–1920 mL (see Supplementary Table 1 for details). The column was washed with PBS pH 7.2 for 6 CV. For the alkaline washed samples, the additional alkaline wash was performed with the 100 mM sodium carbonate, pH 11.0 solution for 6 CV and the subsequent neutralisation with PBS pH 7.2 for 8 CV. We used 100 mM sodium carbonate for pH 10.0 and pH 10.5 buffers. Tris–HCl (100 mM), Glycine–HCl (100 mM), and disodium hydrogen phosphate (100 mM) were used for pH 9.0, 9.5, and 11.5 buffers, respectively. To elute IgG, the 100 mM Glycine–HCl buffer pH 3.2 was applied to the column. The 1.0 M Tris, pH 8.8 buffer was added in advance to the collection tubes to neutralise the eluted solution immediately. The MabSelect SuRe columns used in this study were reutilized after cleaning with 0.5 M NaOH which was a recommended condition for cleaning and sanitization^25^. Each column was not used more than 10 times. All antibody samples were dialyzed to PBS pH 7.2 or 50 mM citrate, 150 mM NaCl, pH 6.3 buffer using a dialysis bag, Slide-A-Lyzer G2, 1,000 Da molecular weight cut-off (Thermo Scientific). Finally, the samples were filtered using a 0.45 µm PES filter GD/X (GE Healthcare). The concentrations of samples were determined with NanoDrop ND-1000 (Thermo Scientific) based on absorption at 280 nm (E^1%^_280_ = 13.7). To compare % recovery of each IgG, the concentrations of IgGs in the representative medium were determined using Bio-Layer Interferometry by BLItz (ForteBIO).

### Size exclusion chromatography (SEC)

SEC was carried out with a TSKgel G3000 SWXL, 7.8 × 300 mm column (Tosoh) connected to a HPLC system (Tosoh). The isocratic elution was carried out with a mobile phase 0.1 M sodium phosphate, 0.1 M sodium sulphate, 0.05% sodium azide pH 6.7 at a flow rate of 1.0 mL/min. The column temperature was kept at 30 °C during analysis. Fifty µL of each 200 µg/mL sample was injected onto the column and analysed with UV detection at the wavelength of 280 nm.

### Microchip electrophoresis (MCE-SDS)

Purities of the IgGs were confirmed by the microchip SDS electrophoresis system Labchip GX II (PerkinElmer). Samples were prepared with HT Protein Express Reagent Kit and HT Protein Express LabChip Kit (PerkinElmer) according to the manual provided by the manufacturer. Dithiothreitol (final concentration 30 mM) or N-ethylmaleimide (final concentration 20 mM) was added to the samples for reducing and non-reducing conditions, respectively. These samples were incubated 5 min at 99 °C (reducing condition) or 70 °C (non-reducing condition). The samples were analysed with the HT Antibody analysis 200 method (14–200 kDa range).

### Irreversible aggregation at high concentration and interaction parameters

The protein A-purified IgG samples were concentrated with ultrafiltration membranes Vivaspin turbo 50,000 Da molecular weight cut-off 15 mL (Sartorius). Particle size distributions were obtained by a dynamic light scattering (DLS) instrument Nanotrac UPA-UT151 (Microtrac). The instrument employs the heterodyne detection system which is suitable for the direct measurement of highly concentrated samples^26^. Particle size distributions of the twofold dilution series of concentrated samples in PBS pH 7.2 were obtained.

As described previously^16^, interaction parameters *k*_*D*_ were generated using DLS data. The diffusion coefficient of each sample was obtained by the Stokes–Einstein Eq.^27^. The interaction parameter was calculated from the concentration dependency of the diffusion coefficient using the following equation.

$${\rm{D }} = {\rm{ }}{{\rm{D}}_0}\left( {1 + {k_D} \cdot {\rm{c}}} \right)$$

where D is the diffusion coefficient obtained from the experimental data, D_0_ is the diffusion coefficient at infinite dilution, and c is the concentration of an IgG (g/mL)^16^.

### Free thiol assay

Free thiols were quantified by 4, 4′ dithiodipyridine (4DTP) (Wako)^28^. The reagent specifically reacts with free thiol groups to generate a by-product which has UV absorption without affecting disulfide bonds. The IgG samples were diluted tenfold with 1 mM EDTA, 0.1 M sodium phosphate buffer (pH 6.0). Fifty µL of 10 mM 4DTP was added to 950 µL sample solution. A dilution series (from 0.00156 to 0.1 mM) of the reduced form of glutathione was used to create the standard curve. All IgG and standard samples were incubated for 10 min at room temperature. UV absorption at 324 nm was measured with a UV spectrometer U-3310 (Hitachi). The free thiols/IgG (mole/mole) values were calculated based on the UV absorption at 280 nm.

### SDS PAGE

The fractions washed out from the protein A column during the washing step were analysed by SDS PAGE using NuPage Bris Tris gel 4–12% (ThermoScientific). The samples were prepared based on the manual provided by the manufacturer. MOPS buffer was used for the electrophoresis. The images were collected by ChemiDoc XRS (Bio Rad) after staining with Instant blue (Expedeon).

### Short-term stability study

IgG-G solution at 10 mg/mL in 50 mM citrate, 150 mM NaCl, pH 6.3 buffer was subjected to the short-term stability test at 40 °C for 3 days. Particle size distributions of tenfold diluted samples were measured by a dynamic light scattering instrument Zetasizer µV (Malvern).

## Results

### Effects of the alkaline wash on yields in protein A purification

**Figure 2:**
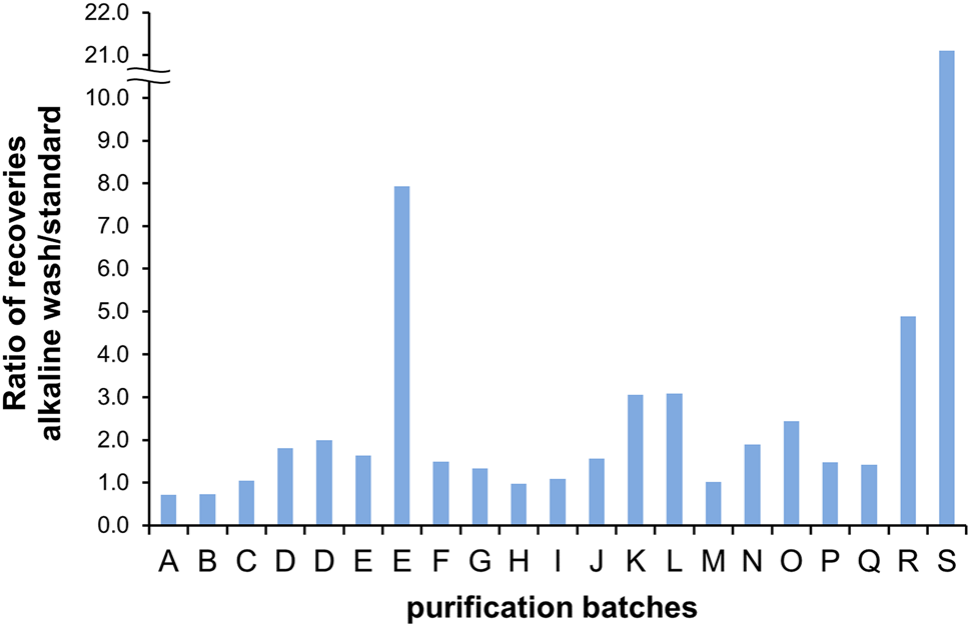
Ratio of recovery during protein A purification. 21 medium batches (19 IgGs) were purified by alkaline wash method and standard method.

The procedure of our protein A purification method with the alkaline wash is shown in Fig. 1. The additional wash with alkaline buffer (pH 9.0–11.0, typically, pH 11.0) and subsequent neutralizing wash step were inserted into the typical protein A protocol. To demonstrate the benefits of the alkaline wash step, 19 human monoclonal IgG 1 antibody clones (17 λ light chain, 2 κ light chain) were purified from 21 medium batches by the standard method or alkaline wash method with a pH 11.0 buffer. Each medium batch was divided into 2 groups to be purified by 2 protocols. To assess the effect of the alkaline method on recoveries of IgGs (amount of purified IgG/amount of IgG in medium), the enhancement ratios in recoveries (recovery of alkaline method/ recovery of standard method) were calculated for each medium batch (Fig. 2, Supplementary Table 2). Surprisingly, 14 out of 21 batches clearly showed improved yields (enhancement ratios > 1.2, i.e. more than 20% increase in yield). In fact, enhancement ratios of more than 3 were observed for 5 out of 21 batches, with the highest value of 21.1 (IgG-S in Fig. 2). Although the yield of IgG-S in the standard method was extremely low (3.0 mg/L, Supplementary Table 2) because of precipitation in the eluate (Supplementary Fig. 1), the alkaline wash method significantly improved the recovery of the difficult-to-purify protein. In contrast, only 2 batches (IgG-A and IgG-B) showed enhancement ratios below 0.9. Thus, the incorporation of alkaline wash into the conventional protein A purification improved %recoveries of most antibodies tested in this study.

### Effects of the alkaline wash on aggregation and purity

Size exclusion chromatography (SEC) and microchip electrophoresis (MCE-SDS) of purified antibodies were carried out to determine if the alkaline wash affected the level of degradation, aggregation or impurities. Data of SEC and MCE-SDS of 6 antibodies (IgG-A, B, C, D, E, and F) are summarized in Table 1. The alkaline wash treatment did not impact aggregation or degradation.

**Table 1:** Effect of alkaline wash on antibody purity.

	SEC (%monomer)		MCE-SDS			
	Standard	Alkaline wash	Reduced (%LC + HC)		Non reduced (%IgG)	
			Standard	Alkaline wash	Standard	Alkaline wash
IgG-A	99.2	98.9	99.0	99.8	89.5	95.1
IgG-B	96.1	97.5	98.4	99.6	97.2	92.8
IgG-C	100	99.8	99.7	99.5	99.0	99.0
IgG-D	100	100	99.4	99.2	97.6	97.5
IgG-E	99.8	99.7	99.6	99.6	97.7	97.8
IgG-F	100	100	98.1	98.9	97.3	89.9

### Irreversible aggregation

**Figure 3:**
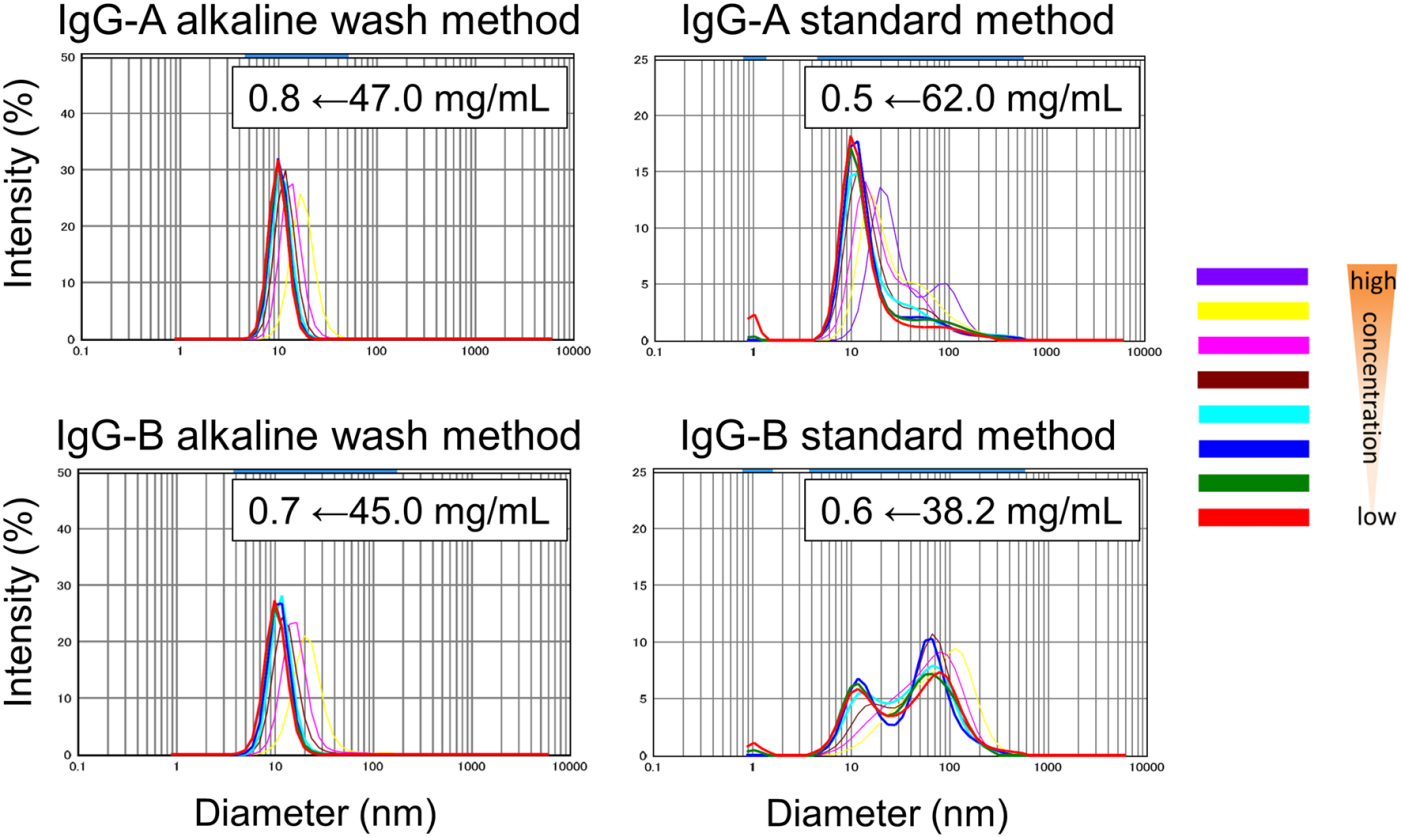
DLS profiles of IgG-A and IgG-B at various concentrations purified by the standard and alkaline wash method.

The purified materials were further analysed to determine whether the alkaline wash affects other parameters or not. First, the ability to concentrate the antibodies was assessed. This parameter is regarded as an important characteristic during the developability assessment to choose one candidate for clinical trials^14^ because highly concentrated formulations have become necessary for certain therapeutic indications. Dynamic light scattering (DLS) is often used to assess the solution behaviours of monoclonal antibodies^14,16^. In this study, particle size distributions of the concentrated antibodies (16 antibodies in Table 2) and their diluents were measured by DLS using Nanotrac UPA-UT151. This instrument utilizes a heterodyne detection system which is more suitable for the direct measurement of highly concentrated samples than a conventional photon correlated spectroscopy^26^. The highest concentration of each concentrated sample is also listed in Table 2. It should be noted that these highest concentrations are not the maximum concentrations. The original target concentration was set to 40–50 mg/mL and some samples were not concentrated because of their poor physicochemical properties. The particle size distributions at various concentrations of IgG-A and IgG-B are shown in Fig. 3 as representatives. The IgG-A and IgG-B samples purified by the standard method showed the aggregate peaks regardless of their concentrations in addition to the monomer peak around 10 nm. Since these aggregate peaks were even observed for diluted samples, the peaks were considered to be derived from irreversible aggregations. In contrast, IgG-A and IgG-B purified with the alkaline wash method did not show any aggregation regardless of their concentrations, indicating that the alkaline wash treatment protected these antibodies from irreversible aggregation. The shift to the larger particle sizes at high concentrations reflects reversible self-interactions of antibody molecules rather than aggregates^16^. The preventive effects of the alkaline wash method on irreversible aggregation were observed for 9 (IgG-A, B, G, H, I, J, N, P and Q) out of 16 antibodies (Table 2). We could not concentrate the conventionally purified IgG-L, O, P, and Q to concentrations of > 20 mg/mL because of poor physicochemical properties. In contrast, these 4 antibodies purified by the alkaline wash method were successfully concentrated to > 30 mg/mL without irreversible aggregation. Thus, alkaline wash treatment protected the antibodies from irreversible aggregation. The other 5 antibodies (IgG-C, D, E, F, and M) showed the reversible association upon concentration irrespective of the purification methods. In fact, 16 antibodies obtained from the alkaline wash treatment were successfully concentrated without any detectable irreversible aggregation.

### Quantification of free thiol groups

To explore the mechanism of how the alkaline wash treatment prevented antibodies from irreversible aggregation, quantification of free thiol groups was carried out using the thiol-reactive agent 4, 4′ dithiodipyridine^28^. As shown in Table 2, free thiols of 15 IgGs were measured. The 15 antibodies include 8 antibodies (IgG-A, B, G, H, I, J, N and Q) which showed the irreversible aggregation when purified by the standard method (Fig. 3, Table 2). Among these 8 irreversibly-aggregated antibodies, the 6 antibodies (IgG-A, B, G, H, I, and J) purified by the standard method showed high free thiols/antibody molecule values (Table 2). On the contrary, alkaline-washed IgG-A, B, G, H, I, and J samples clearly showed much lower free thiols/antibody values. Since high levels of free thiols are considered one of the causes of irreversible aggregations for IgGs^29^, the protective effect of the alkaline wash on irreversible aggregation (Fig. 3) could be ascribable to the accelerated generation of thiolate ions serving as reactive species in thiol-disulfide exchange and disulfide formation at a pH above the pKa of thiol groups^30,31^. The other 7 antibodies (IgG-C, D, E, F, L, M, and O) which did not show the irreversible aggregation with the standard purification (Table 2) showed much lower free thiols/antibody molecule values than the irreversibly aggregated antibodies (IgG-A, B, G, H, I, and J) purified by the standard method. This fact also supports the notion that the main mechanism by which the alkaline wash prevents the irreversible aggregation is by reducing the number of free thiols.

**Table 2:** Effects of alkaline wash on physicochemical characteristics.

Antibody	Aggregation by DLS				Free thiol		Self-interaction, *kD*	
	Standard		Alkaline		Standard	Alkaline	Standard	Alkaline
	Concentration mg/mL	Mode	Concentration mg/mL	Mode	mol/mol	mol/mol	mL/g	mL/g
IgG-A	62.0	Irreversible	47.4	Reversible	0.30	0.04	n.d	− 11.3
IgG-B	38.2	Irreversible	44.6	Reversible	1.31	0.04	n.d	− 16.9
IgG-C	49.2	Reversible	52.0	Reversible	0.16	0.09	− 25.6	− 8.2
IgG-D	45.9	Reversible	37.0	Reversible	0.16	0.07	− 19.3	− 8.7
IgG-E	49.2	Reversible	67.3	Reversible	0.16	0.03	− 14.5	− 8.5
IgG-F	30.7	Reversible	26.6	Reversible	0.13	0.05	− 36.6	− 16.2
IgG-G	44.7	Irreversible	46.6	Reversible	0.15	0.08	n.d	− 7.0
IgG-H	42.2	Irreversible	48.4	Reversible	1.36	0.06	n.d	− 29.8
IgG-I	42.6	Irreversible	43.8	Reversible	0.78	0.10	n.d	− 39.7
IgG-J	38.9	Irreversible	42.6	Reversible	0.45	0.20	n.d	− 35.4
IgG-L	15.4	Reversible	42.5	Reversible	0.12	0.05	− 33.8	− 37.1
IgG-M	51.9	Reversible	50.9	Reversible	0.12	0.09	− 6.6	− 8.5
IgG-N	12.9	Irreversible	48.6	Reversible	0.06	0.00	n.d	− 10.5
IgG-O	7.1	Reversible	42.2	Reversible	0.01	0.02	− 46.5	− 19.5
IgG-P	5.0	Irreversible	38.6	Reversible	n.d	n.d	n.d	− 13.4
IgG-Q	17.5	Irreversible	42.3	Reversible	0.05	0.04	n.d	− 26.8

n.d., not determined.

### Self-interaction of IgG molecules

Next, the self-interactions of the antibodies were assessed (Table 2, Fig. 3). The self-interaction of IgG molecules is considered as an important factor in the development of a stable highly concentrated formulation because the propensity of self-interaction is closely related to a high viscosity and an accelerated aggregational rate^16,32–34^. The concentration-dependent DLS analysis is often employed to evaluate the tendencies of IgGs to self-interact during the developability assessment. The interaction parameter *k*_*D,*_ which is calculated from DLS data measured at various concentrations, is a key parameter to judge if the intermolecular interaction is repulsive or attractive^16,32^. Lehermayr et.al. reported that the *k*_*D*_ values of ca. − 9 mL/g corresponds to no self-interaction^35^ for monoclonal antibodies which is in close agreement with the results of other groups^16,33,34^. This result indicates that a *k*_*D*_ value larger than − 9 mL/g could be assumed to be a repulsive intermolecular interaction. On the contrary, a *k*_*D*_ value smaller than − 9 mL/g could be considered as an attractive interaction. The repulsive self-interaction of antibody is preferable for therapeutic use, because it is likely to lead to a lower viscosity and a slower aggregation rate^16,32–34^.

In this experiment, the same procedure was employed as the experiment for irreversible aggregation (Fig. 3, Table 2) to obtain *k*_*D*_ values. The comparison of the interaction parameters of the 7 antibodies (IgG-C, D, E, F, L, O, and M) which did not irreversibly aggregate upon concentration regardless of the purification methods clearly showed that the alkaline wash treatment improved the *k*_*D*_ values except for IgG-L and IgG-M. Table 2 shows that the *k*_*D*_ values of IgG-C, D, E, F, O and L purified by the standard method were smaller than − 9 mL/g, suggesting that these antibodies tend to associate attractively. In contrast, the 3 antibodies (IgG-C, D, and E) purified using the alkaline wash method showed *k*_*D*_ values above − 9 mL/g, indicating that these samples had a slightly repulsive self-interaction. Surprisingly, the alkaline wash method significantly improved the tendency of the protein A purified samples to self-associate. As free thiols of IgG-C, D, E were also decreased by washing with the alkaline buffer, free thiols could be involved in the improvement in the reversible self-association tendencies. In addition to a reduction in free thiols, an alkaline wash could remove other impurities that promote self-association which might be another reason of this effect. The interaction parameters of IgG-A, B, G, H, I, J, N, O, P and Q were calculated only for the samples purified by the alkaline wash since the presence of the aggregated peaks could hamper the analysis (Table 2).

### Elution of impurities by alkaline wash treatment

**Figure 4:**
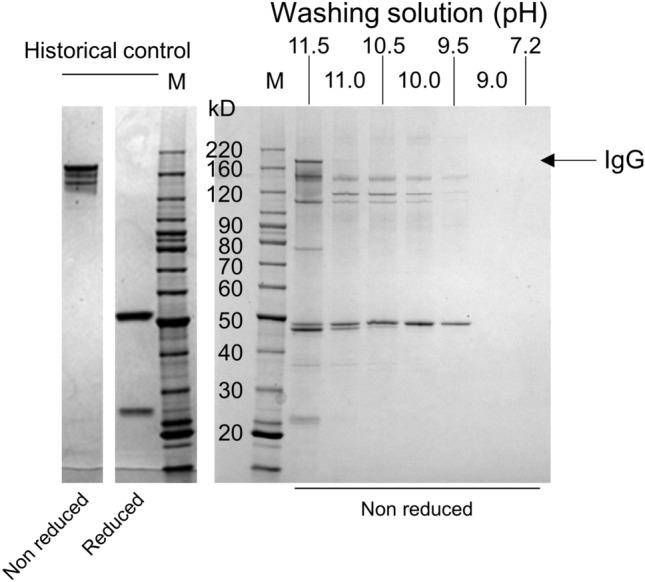
SDS PAGE analysis of washing fraction of IgG-C. M means a molecular marker. Molecular masses are indicated.

To investigate the effects of the alkaline wash treatment on removing impurities, SDS PAGE analysis of IgG-C was carried out under non-reducing conditions. The fractions washed out from the protein A column during the washing step with several alkaline buffers (pH 9.0–11.5) were collected and subjected to SDS PAGE (Fig. 4). The pH 7.2 and 9.0 buffers did not elute obvious impurities, however, buffers with a pH above 9.0 eluted several bands. The pH 11.5 buffer eluted the target antibody, but no eluted IgG-C was observed in other conditions. The origin of the bands slightly above 20 kDa and slightly below 40 and 50 kDa is unknown as they did not match the light chain and the heavy chain of IgG-C, respectively (Fig. 4). The difference in the conditions for SDS PAGE (non-reducing or reducing) often affects the electrophoretic mobility of proteins, therefore, we could not clearly determine the origin of these bands. We have not been able to conclude yet that other bands located in 70–80, 100–120, and 120–160 kDa are IgG-C with free thiols or host cell proteins. Another group previously reported that the hamster host cell protein phospholipase B-Like 2 showed at least 3 bands in the range of 20–60 kDa in their SDS PAGE analysis^36^. As the molecular weight range of the hamster protein is very close to those of IgGs, further studies are needed to be done to identify the origin of these bands eluted by the alkaline wash. Considering that the SDS-PAGE and SEC profiles of the purified materials only contained the species related to general IgGs (data not shown), these data strongly suggested that the alkaline wash eluted these impurities regardless of their origins without affecting protein A-IgG interaction in the pH range of 9.5–11.0 (Fig. 4).

### Short-term stability test

**Figure 5:**
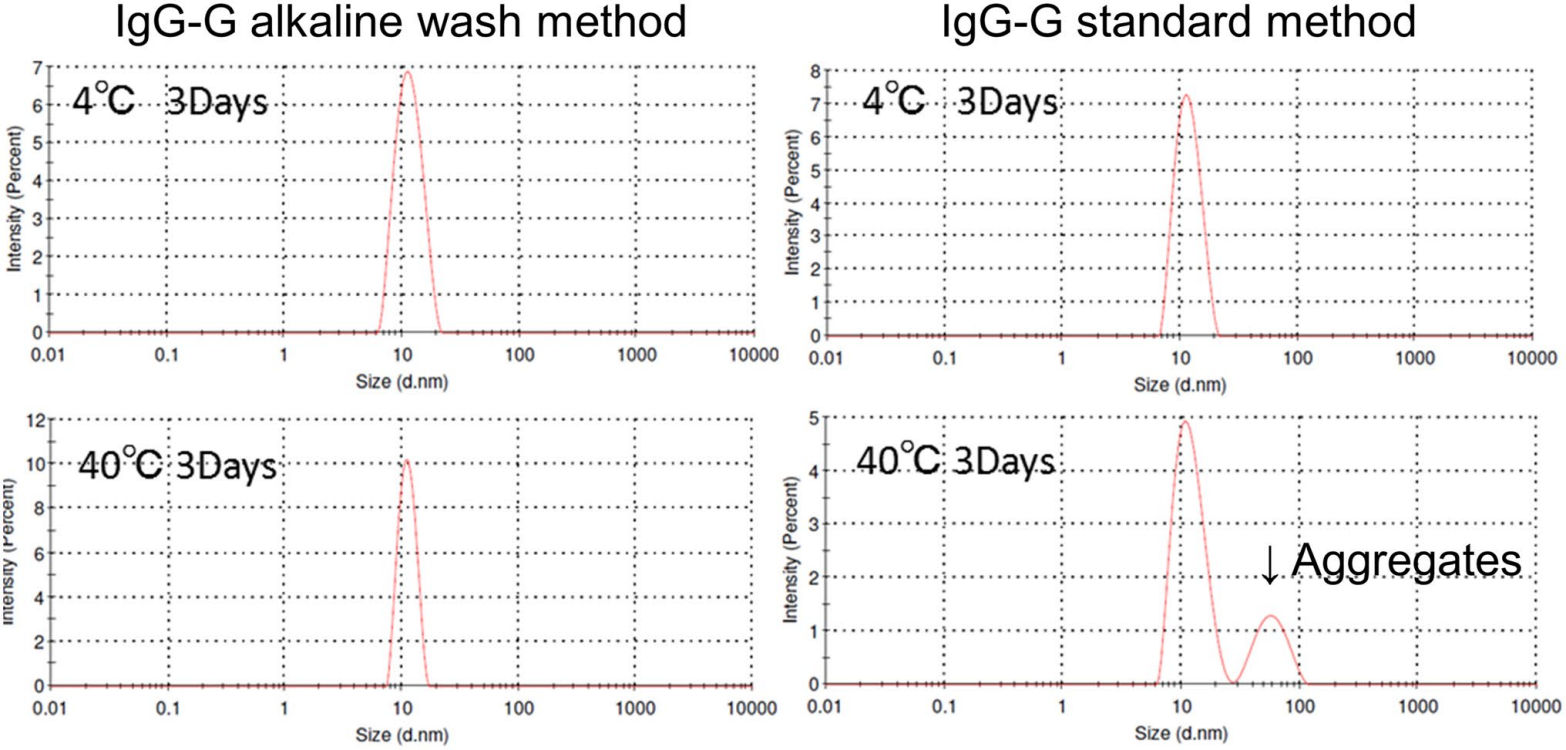
DLS profiles of short-term stability test samples of IgG-G.

Accelerated stability tests at 40 °C are frequently used to estimate the long-term stabilities of antibodies at a target storage temperature like 2–8 °C^32^. We carried out a short-term stability test to determine whether irreversible aggregations are formed during storage. The ~ 10 mg/mL IgG-G samples purified by the standard and alkaline wash method were stored in 50 mM citrate, 150 mM NaCl, pH 6.3 buffer at 40 °C for 3 days. The collected samples were diluted tenfold with the same buffer and analysed by DLS to obtain particle size distribution (Fig. 5). The IgG-G sample purified by the alkaline wash method did not show any aggregation after 3 days at 40 °C. In contrast, IgG-G purified using the standard method showed an aggregational peak in DLS analysis. By using the alkaline wash method, the short-term stability of IgG-G was improved.

## Discussion

It has been about 50 years since Hjelm et. al. clearly demonstrated the concept of affinity purification of IgG by using immobilized protein A resins^37^. Since then, considerable progress has been made in the field of protein A affinity purification such as novel protein A resins with modified sequences to attain better alkaline resistance and improved binding capacities^4^. In addition, researchers have been studying the effects of several wash and elution buffers to maximize yields and minimize impurities^17,19,20,22,23^.

Regardless of these extensive efforts, the effects of different buffer conditions at each step of protein A purification on the physicochemical character of IgGs were not fully elucidated. In this study, we showed our new alkaline wash method had multiple beneficial effects on the physicochemical properties of purified antibodies; i.e., the method protected IgGs from irreversible aggregation (Fig. 3), reduced attractive self-interactions (Table 2), decreased free thiols (Table 2), and significantly improved recoveries (Fig. 2) and short-term stability (Fig. 5). As far as we know, there is no similar example demonstrating that such a simple insertion of an additional washing step into the conventional protein A method improved several physicochemical properties as well as yields of purified IgGs at the same time.

In addition to acid solutions, alkaline buffers have been frequently used as elution buffers to break strong antibody-antigen interactions during immunoaffinity purifications^38^. However, the effects of alkaline buffers on protein A-based IgG purification have not been investigated except for a few reports^19,22–24^. In this study, the alkaline washing buffers (pH 9.5–11.0) did not elute our IgG (Fig. 4) in accordance with the results of other groups that used similar basic washing buffers (pH 9.0–10.4) ^19,22,23^. Surprisingly, our data revealed that the highly basic buffer with a pH 11.0 did not elute IgG-G (Fig. 4). The pH 11.5 buffer partially eluted IgG-G, suggesting that the threshold could exist between pH 11.0 and 11.5 (Fig. 4). The histidine residue at the position of 435 in IgG1 heavy chain is considered as one of the most important residues in the protein A-IgG interactions^39,40^and the acidic environment can induce the protonation of the histidine residue to impair their interaction^40^. If this mechanism is a main rationale for the acid elution, it is reasonable to assume that the alkaline buffers did not elute the IgG molecules because basic buffers would not affect the charge state of the histidine. The partial elution of IgG observed at high pH 11.5 could be attributable to structural changes also suggested for the elution mechanism by acidic buffers^40–42^.

As the alkaline buffer did not elute the IgGs, we initially expected recoveries of the IgGs from cultured medium purified by alkaline method to be similar to those purified by the standard method. However, the alkaline wash treatment significantly increased the yields of many IgGs against our expectations (Fig. 2). This is a very important discovery because the yield is one of the most important parameters during developability assessment, process development, and manufacturing. Wang et.al. also compared the yields between the alkaline wash (pH ~ 10) and standard method for 3 antibodies, however, they found comparable yields between the two methods^23^. The limited numbers of purification batches in their study could be one of the reasons for the different conclusions between their study and ours because we purified 19 antibodies from 21 cultured medium batches and confirmed obvious enhancements in recoveries for 14 out of 21 batches (Fig. 2). It is also possible that isotypes could impact the results. Wang et.al. used 1 IgG4 and 2 IgG1. The isotypes of the light chains were not specified^23^. In our study, 19 IgG1 antibodies (17 λ and 2 κ light chains) were used. In addition, culturing conditions and host cells could also impact the extent of yield enhancement effect. The alkaline wash improved the yields of two batches of IgG-E with different degrees (Fig. 2). The recovery enhancement ratios of some antibodies were higher than 2, indicating that the recoveries of these antibodies purified by the standard purification were at least lower than 50% (Fig. 2 and Supplementary Table 2). Our data suggest that this method is especially useful for these antibodies which are not easily purified by the standard method. The detailed mechanism for the yield enhancement effect of the alkaline wash remained unclear at present. As both the alkaline method and control method employed the same procedures in the capture step and elution step, the differences in the amount of IgGs bound to protein A and the efficiency in stripping antibodies from protein A in the elution step must be negligible. The improvement in physicochemical characteristics of antibodies by the alkaline method is possibly related to the underlying mechanism for the yield enhancement. However, no clear relationship between the yield enhancement (Fig. 2) and the physicochemical characteristics (Table 1 and 2) was derived from our results except for the tendency that the free thiols/antibody values were somewhat inversely correlated to the yield enhancement. All antibodies with free thiol/antibody values of less than 0.2 when purified by the standard method showed the yield enhancement values of higher than 1.2 (Fig. 2 and Table 2). The detailed mechanism for this correlation is still unclear, however, it is possible that the fact that the alkaline wash decreased the free thiol/antibody values (Table 2) is involved. Regarding effective pH range, our data using IgG-C and washing solutions with various pH showed the yield was enhanced at pH above 9.5–10 and did not vary significantly in the range of 9.5–11.5 (Supplementary Fig. 2). The reason for the yield enhancement by the alkaline wash needs to be explored more in the future to optimize the conditions of alkaline wash for various antibodies.

The propensity of an IgG to aggregate is also an important parameter because aggregates could lead to severe adverse effects by inducing immunogenicity^10–12^. Therefore, even though a new purification method has multiple benefits, the technology is not widely applicable if the aggregation rate is increased by the new method. Our SEC analysis showed that the insertion of the alkaline wash step did not increase the aggregation of freshly purified samples (Table 1) in accordance with a previous report^23^. However, we discovered that the differences in aggregation between the alkaline-washed samples and their corresponding controls appeared only after concentration by ultrafiltration to the final concentration of several tens of mg/mL (Table 2 and Fig. 3). The 10 antibodies including IgG-A and IgG-B purified by the standard method irreversibly aggregated after the concentration step (Table 2 and Fig. 3). In contrast, the alkaline-washed antibodies did not show any detectable aggregation by DLS. This protective effect is very important for therapeutic antibodies because the particles within the detectable size range for DLS (from nm to single digit µm) are often designated as “subvisible particles” and were suggested to be highly related to immunogenicities^12,43^. This alkaline wash method also improved aggregation of IgG-G in the short-term stability test (Fig. 5). Effects of the alkaline wash on stability during storage are needed to be carefully examined using several antibodies in the future because we carried out the short-term stability test only with IgG-G.

The high free thiols/IgG values of the control samples could be one of the more likely causes for the irreversible aggregation as another group reported previously^29^. As the formation rate of disulfide bonds depends on the concentration of thiolate ions^30,31^, the aggregation was probably observed only after the ultrafiltration. Thus, the reduction in the numbers of free thiols by the alkaline wash was suggested to be a plausible mechanism for the protection of IgGs from irreversible aggregation during concentration. In fact, the decrease in free thiols correlated well with the preventive effect (Table 2). Still, the antibodies IgG-O and IgG-Q with the low thiols/antibody values showed irreversible aggregation only when they were purified by the standard method, suggesting that other mechanisms for the prevention of irreversible aggregation by the alkaline wash also could exist However, we have not obtained any data regarding the exact positions of the free thiols in these IgG molecules so far. It is reported that the disulfide bond that is most susceptible to reduction is the interchain bond linking the light and heavy chains^44^. Therefore, the cysteines of this interchain disulfide were expected to be involved in the irreversible aggregation in our study. In contrast to this report, our non-reducing MCE-SDS analysis suggested that most of the interchain disulfide bonds of our antibodies were formed regardless of their free thiol/antibody values (Tables 1 and 2). Therefore, interchain disulfide bonds could not be responsible for the high free thiol/antibody values. The exact locations of free thiols involved in the protective effect against the irreversible aggregation should be determined by non-reduced peptide mapping analysis in the future.

A few groups studied alkaline wash procedures mainly to reduce impurities such as host cell proteins and DNA^19,22,23^. Wang et al. reported that an alkaline wash with a pH 10.4 buffer decreased host cell proteins by one fifth compared to the conventional procedure. Our data also indicated that impurities were eluted by the alkaline wash without eluting the target IgG (Fig. 4) in the pH range of 9.0–11.0. However, we have not been able to identify the origin of these impurities. Because the molecular weights of host proteins derived from CHO cells are close to those of antibody components^36^, the identity of bands observed in our SDS PAGE analysis (Fig. 4) should be carefully determined in the future. Besides the beneficial effects induced by the alkaline wash, the basic condition could impose undesired effects on target antibodies. Especially, deamidation is known to occur in basic conditions with a higher rate than in acidic or neutral conditions^45,46^. Deamidation should be carefully considered because it possibly leads to the loss of activity^47^. Wang et.al. reported that the wash with the pH ~ 10 buffer had no impact on deamidation as determined by peptide mapping coupled to mass spectrometry^23^. Similarly, we also confirmed the comparable peptide mapping results between the alkaline washed and control samples (Supplementary Fig. 3), suggesting that the alkaline wash did not induce any additional chemical modifications. In addition to the chemical integrity, Wang et.al. employed circular dichroism to confirm that the secondary structures of antibodies purified by the alkaline wash method were comparable to the control samples^23^. The structural integrities of the alkaline-washed antibodies were also supported by our melting temperature data measured by means of a hydrophobic dye (data not shown). ELISA carried out by Wang et.al.^23^ and our in-house cell-based assays showed the comparable activities of the alkaline-washed and conventionally purified antibodies (data not shown), indicating that the alkaline wash did not alter the activities of antibodies. Our surface plasmon resonance analysis also showed that the alkaline wash did not affect the binding abilities of the IgGs to the FcγRIIIA receptor which is responsible for the antibody dependent cellular cytotoxicity (data not shown). We suggest that the adverse effects of alkaline solution are limited because the washing time is very short (6 min). The alkaline wash is also less likely to cause a significant damage to the protein A resin MabSelect SuRe used in this study because the resin has better alkaline resistance and retains more than 85% dynamic binding capacity even after exposed to a harsher cleaning condition (0.1 M NaOH, 150 cycle × 15 min)^25^ than our alkaline wash.

In conclusion, we found a novel alkaline wash method possessing several benefits such as the enhancement in yields, protection from irreversible aggregation, decrease in free thiols, improvement in self-interaction, short-term stability, and reduction in impurities. Furthermore, the procedure is very simple and versatile so that researchers can easily apply this method during small-scale purifications at the research stage as well as larger-scale manufacturing at the development stage. Nowadays, the development of antibody therapeutics has been becoming more competitive than before; many companies have programs with identical targets^1^. In this situation, the time for sample preparation and process development is minimized to achieve faster research and development. At the research stage, alkaline wash can help to reveal the actual physicochemical characters of candidate antibodies. For example, 10 antibodies (IgG-A, B, G, H, I, J, N, O, P, and Q) were protected from irreversible aggregations when they were prepared by the alkaline wash method. If only the conventional protein A purification were available, these antibodies may have been dropped early on from candidates for therapeutic use because of their poor physicochemical properties. Thus, researchers can evaluate the actual character of antibodies and retain more candidates. The method can contribute to the development stage as well. In the conventional manufacturing, two polishing columns follow the protein A capture step. It is possible that optimization of the protein A step with an alkaline wash could reduce the number of polishing steps in the future. This will enable faster development with lower cost. To achieve this goal, in addition to impurities such as host cell proteins and aggregated antibodies, other impurities should be further examined in terms of viral clearance, leached protein A, and DNA.

## Supplementary Information

Supplementary Information.

## Data Availability

All data generated or analyzed during this study are included in this published article.
