# Publisher Correction: Washing with alkaline solutions in protein A purification improves physicochemical properties of monoclonal antibodies

**DOI:** 10.1038/s41598-021-92414-6

**Published:** 2021-07-22

**Authors:** Yuichi Imura, Toshiaki Tagawa, Yuya Miyamoto, Satoshi Nonoyama, Hiroshi Sumichika, Yasuhiro Fujino, Masaya Yamanouchi, Hideo Miki

**Affiliations:** 1 Sohyaku. Innovative Research Department, Mitsubishi Tanabe Pharma Corporation, Yokohama, Japan; 2 Sohyaku. Innovative Research Department, Mitsubishi Tanabe Pharma Corporation, Fujisawa, Japan; 3 Present Address: Development Department, Tanabe Research Laboratories U.S.A. Inc., San Diego, USA; 4 Present Address: Research Department, Tanabe Research Laboratories U.S.A. Inc., San Diego, USA

Correction to: *Scientific Reports* 10.1038/s41598-021-81366-6, published online 19 January 2021

The original version of this Article contained extensive errors in the Reference list.

Reference 26 was incorrectly split into Reference 26 and Reference 27.

“Trainer, M. N. & Freud, P. J. High-Concentration Submicron Particle Size Distribution by Dynamic Light Scattering Power spectrum development with heterodyne technology advances biotechnology and nanotechnology measurements.”

now reads:

“Trainer, M. N. & Freud, P. J. High-Concentration Submicron Particle Size Distribution by Dynamic Light Scattering Power spectrum development with heterodyne technology advances biotechnology and nanotechnology measurements. Microtrac, Inc. Application Note SL-AN-05 Rev B. (2009).”

As a result, References 27–47 were incorrectly listed as References 28–48.

The original Article has been corrected.

