# Supplementary material for: Washing with alkaline solutions in protein A purification improves physicochemical properties of monoclonal antibodies
Source: Sci Rep. 2021 Jan 19;11:1827. doi: 10.1038/s41598-021-81366-6 (PMC7815873; doi:10.1038/s41598-021-81366-6)
Supplement: Supplementary file 1 — Supplementary Information. [file 41598_2021_81366_MOESM1_ESM.pdf]

Washing with alkaline solutions in protein A purification improves physicochemical properties of monoclonal antibodies

Yuichi Imura, Toshiaki Tagawa, Yuya Miyamoto, Satoshi Nonoyama, Hiroshi Sumichika, Yasuhiro Fujino, Masaya Yamanouchi, and Hideo Miki

Supplementary Table 1. Summary data for protein A purification in this study.

Antibody	Volume of cultured medium, mL (Amount of IgG purified/1 L medium, mg/L)		Ratio of recovery Alkaline/Standard
	Standard	Alkaline wash	
IgG-A	1450 (55.2)	900 (39.6)	0.72
IgG-B	1920 (23.9)	1800 (17.4)	0.73
IgG-C	480 (51.3)	1400 (53.1)	1.04
IgG-D (batch 1)	1400 (11.1)	500 (20.0)	1.80
IgG-D (batch 2)	700 (91.3)	700 (91.3)	1.99
IgG-E (batch1)	485 (33.0)	1400 (53.7)	1.63
IgG-E (batch2)	1000 (4.2)	900 (33.3)	7.93
IgG-F	1400 (9.4)	500 (14.0)	1.48
IgG-G	900 (31.2)	900 (41.6)	1.33
IgG-H	950 (96.3)	950 (94.5)	0.98
IgG-I	950 (31.4)	950 (34.3)	1.09
IgG-J	950 (22.3)	950 (34.7)	1.56
IgG-K	900 (5.0)	900 (15.3)	3.07
IgG-L	900 (8.7)	900 (26.9)	3.10
IgG-M	900 (60.2)	900 (61.1)	1.01
IgG-N	500 (11.6)	1400 (22.0)	1.90
IgG-O	500 (3.4)	1400 (23.2)	2.44
IgG-P	500 (8.8)	1400 (12.9)	1.46
IgG-Q	500 (9.2)	1400 (13.1)	1.43
IgG-R	500 (0.8)	1400 (3.9)	4.82
IgG-S	600 (3.0)	1300 (63.2)	21.07

Supplementary Table 2. IgG concentration in the medium and recovery ratio of 6 representative IgGs.

Antibody	Purification method	Amount of IgG purified/1 L medium (mg/L)	IgG concentration in medium (Blitz) (mg/L)	Recovery (%)	Ratio of recovery Alkaline/Standard
IgG-C	Standard	51.3	60.5	85	1.04
	Alkaline	53.1		88	
IgG-D	Standard	45.8	72.3	63	1.99
	Alkaline	91.3		126	
IgG-E (batch 1)	Standard	33.0	86.6	38	1.63
	Alkaline	53.7		62	
IgG-G	Standard	31.2	34.8	90	1.33
	Alkaline	41.6		120	
IgG-M	Standard	60.2	68.7	88	1.01
	Alkaline	61.1		89	
IgG-S	Standard	3.0	75.9	4	21.07
	Alkaline	63.2		83	

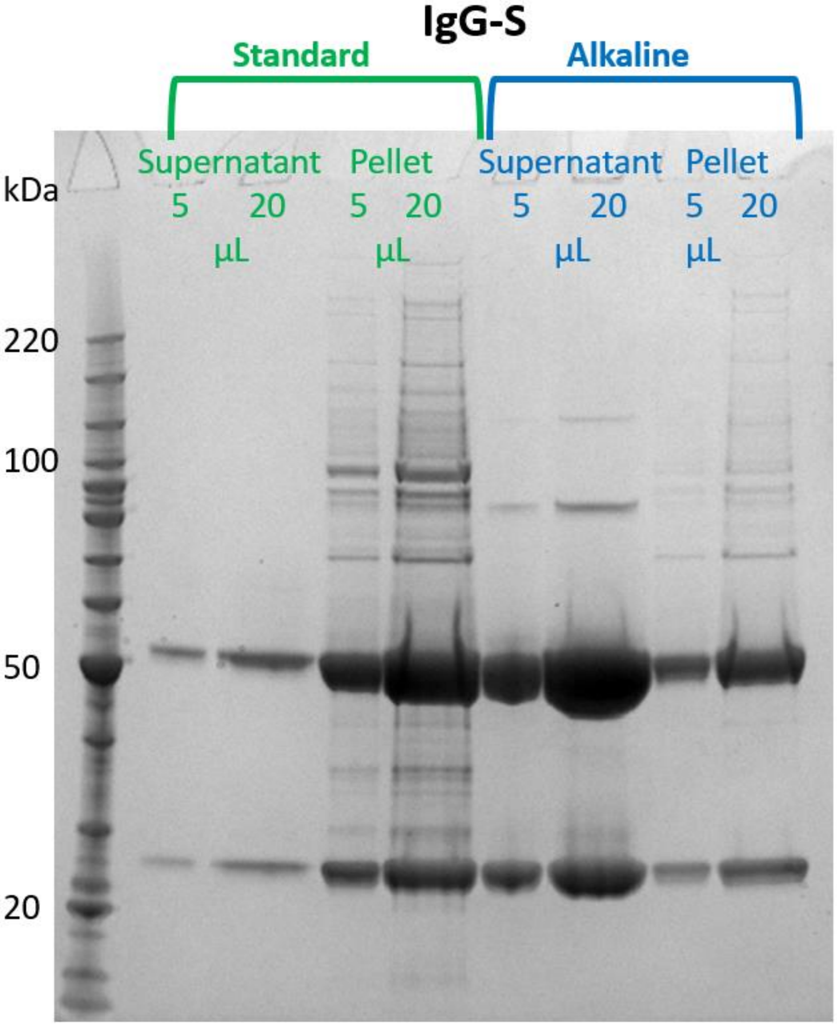

Supplementary Figure 1. SDS PAGE of eluted and precipitated fractions of IgG-S.

IgG-S was purified from culture medium by the chromatography system AKTA explorer 100 (GE Healthcare) and the HiTrap MabSelect SuRe (GE Healthcare, 5 mL of column volume, CV). After equilibrating the column with PBS pH 7.2 (Life Technologies), the cultured medium was applied to the system. The volumes of cell culture medium used for purification were 600 and 1300 mL for the standard and alkaline wash protocols, respectively (Supplementary Table 1). The flow rate was maintained at CV/min. The column was washed with PBS pH 7.2 for 6 CV (standard protocol). For the alkaline wash protocol, the additional alkaline wash was performed with the 100 mM sodium carbonate, pH 11.0 solution for 6 CV and the subsequent neutralisation with PBS pH 7.2 for 8 CV. To elute the IgG-S, the 100 mM Glycine-HCl buffer pH 3.2 was applied to the column. The 1.0 M Tris, pH 8.8 buffer was added in advance to the collection tubes to neutralise the eluted solution immediately. The elution solution was centrifuged at 10000 ×*g* for 10 min to separate precipitates from supernatant. The precipitated pellet was resuspended with 30 mL of 8 M urea and was subjected to SDS PAGE.

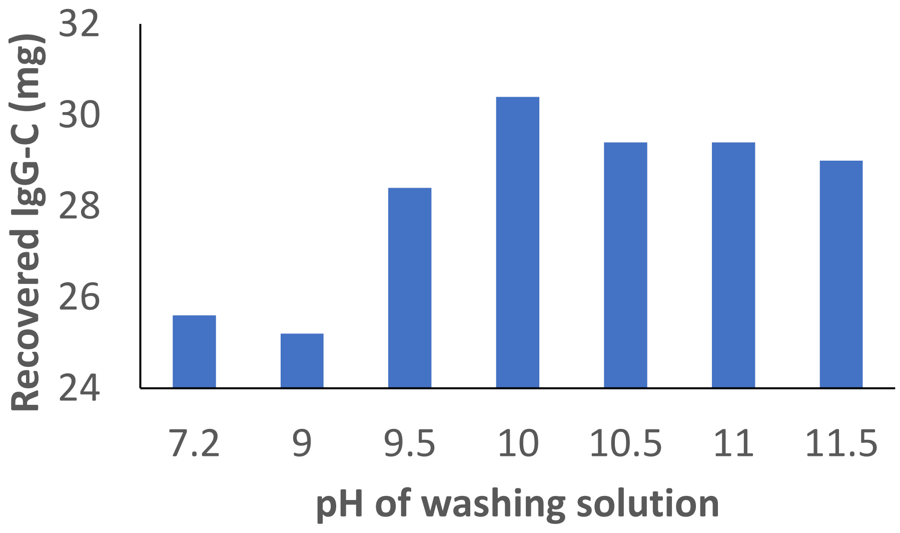

Supplementary Figure 2. Effects of pH of washing solution on recovery of IgG-C.

The culture medium expressing IgG-C was purified by protein A column (ToyoScreen AF-rProtein A HC-650F, 5.0 mL). In the washing step after the capture step, washing buffers with various pH (7.2-11.5) were tested. 50 mL of the culture medium were injected for each condition. To prevent carryover, the column was cleaned with 0.5 M NaOH between purifications as recommended by the manufacturer.

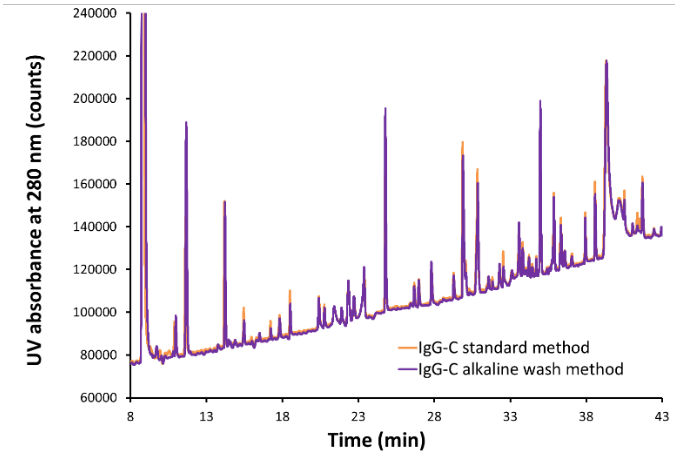

Supplementary Figure 3. Tryptic peptide mapping of IgG-C.

UV chromatograms of IgG-C purified with standard and alkaline wash method are coloured in orange and purple, respectively. IgG-C purified by the alkaline wash and standard method were denatured in the in the presence of guanidine HCl (ca. 5 M), followed by reduction with dithiothreitol (ca. 50 mM) at 37 ºC for 2 hours. The generated free thiols were then alkylated with iodoacetamide (ca. 100 mM). After the buffer exchange to 2 M urea, the antibody was enzymatically digested with trypsin overnight. The tryptic peptides were separated by reversed phase using an ACQUITY UPLC Peptide BEH C18 column, 1.7 µm, 2.1 mm 150 mm (Waters). The column was connected to a Waters ACQUITY UPLC coupled to Waters Synapt tandem mass spectrometer. A conventional binary gradient with 0.1% formic acid in water (mobile phase A) and 0.1% formic acid in acetonitrile (mobile phase B) was used for separation of the tryptic peptides. The total flow rate was maintained at 0.2 mL/min and the percentage of mobile phase B was linearly increased from 1% to 40% in 60 minutes. UV absorbance was monitored at 215 nm and mass spectra of the eluent from the column was concomitantly obtained. The mass spectrum was analysed with BiopharmaLynx software (Waters).
